# Application of the Balance Model in the Analysis of Factors Responsible for Depressive Disorders among Women in the COVID-19 Pandemic

**DOI:** 10.3390/ijerph19127361

**Published:** 2022-06-15

**Authors:** Ewa Dobiała, Anna Gulczyńska, Rafał Małecki, Polina Efremova, Joanna Ławicka, Ewa Karmolińska-Jagodzik, Ivan Kirillov

**Affiliations:** 1 Positive Psychotherapy Center, ul. Leśna Osada 5, 64-100 Leszno, Poland; edobiala@gmail.com; 2 Faculty of Educational Studies, Adam Mickiewicz University, ul. Szmarzewskiego 89, 60-568 Poznan, Poland; ekarmoli@amu.edu.pl; 3 Department of Angiology, Hypertension and Diabetology, Wroclaw Medical University, Center for Research and Development, Voivodeship Specialty Hospital, 51-124 Wroclaw, Poland; rmalecki@gazeta.pl; 4 World Association for Positive and Transcultural Psychotherapy, Luisenstrasse 28, 65185 Wiesbaden, Germany; poromesh@gmail.com (P.E.); ivan@positum.net (I.K.); 5 Fundacja Prodeste, ul. Pierwiosnkowa 6, 53-225 Wrocław, Poland; j.lawicka@prodeste.pl

**Keywords:** positive psychotherapy, balance model, depression, COVID-19 pandemic, women

## Abstract

The COVID-19 pandemic has significantly affected the lives and mental health of people around the world, and it has become clinically essential to define risk factors in order to provide adequate prevention and support. The aim of the study was to describe coping strategies in Polish women related to the COVID-19 pandemic using the balance model, one of the most important concepts of positive psychotherapy (PPT after Peseschkian since 1977). The analysis included 735 women at the mean age of 39.61 years. The survey was conducted using the questionnaire form on the website. Based on Beck’s depression test, depressive disorders were disclosed in 32.65%, and both the presence and severity of depressive syndromes were inversely correlated with age. Using a cluster analysis, three adaptation strategies could be identified, related to the different prevalence of depressive disorders. Relationships proved the most crucial area of the balance model, responsible for the effectiveness of the coping strategy. Based on the obtained results, it has to be concluded that preventive measures should primarily concern women aged < 25 years old and focus on strengthening the relationships area.

## 1. Introduction

Coronavirus (SARS-CoV-2) infections have become one of the significant challenges of the 2020/2021 world. In response to a problematic epidemiological, economic and social situation [1], both the exacerbation of chronic diseases and an increase of adaptive depressive reactions, stress symptoms and mental disorders have been observed clinically.

Studies performed during the epidemic showed that depressive disorders were diagnosed more often in the group of women than men, which was verified by the results of many meta-analyses [2,3]. The authors also emphasize the significance of the higher incidence of depression, particularly among young women [4,5]. Moreover, a group of adolescents and students have experienced distinct stressors, faced not only in mental health, but also in wider social life and cognitive functions, including learning. Studies show [6,7,8] that a group of adolescents and students have experienced distinct stressors, faced not only in mental health, but also in wider social life and cognitive functions, including learning.

The global community of clinicians and scientists is being engaged in searching for the concepts that allow for better understanding of the changes occurring nowadays, as well as defining risk factors, in order to implement adequate support and prevention. It seems important to identify the factors that protect against the development or exacerbation of mental disorders, which can be used by an individual on his own.

Positive psychotherapy (PPT, since Pesechkian, 1977) represents a humanistic–psychodynamic approach that integrates the humanistic concept of personality, psychodynamic understanding of conflict dynamics, systemic reflection that includes transgenerational and transcultural aspects, cognitive–behavioral elements and strategic thinking, including the one based on a humanistic resource-based and goal-oriented five-step psychotherapeutic process [9]. The assumptions of the PPT are grounded in a transcultural mental model, in which the forms of unified human behaviour are shaped at individual, family and cultural levels [10].

The balance model, a basic tool of PPT, allows for recognizing the dynamics of conflicts and identifies resource areas, as well as gives an insight into the possible course of development of the adaptive response of a patient. It reflects the distribution of a person’s daily life activity in four areas: body, relationships, productivity and creativity (fantasy, spirituality) [11]. Time and energy invested in each of these dimensions represents an individual feature, determined by a particular pattern of biological, psychodynamic, systemic and transcultural variables. During actual conflict (difficult situation), changes in the abovementioned dimensions become more pronounced. Clinically, both “escape to” a given area (e.g., escape to the area of the body or fantasy) and “escape from” a given area (e.g., escape from the body, contact or productivity) can be observed. The above dynamics occur in response to the experienced threat of a lack of satisfaction in the area of basic emotional needs (Primary Capacities, Table 1).

During their lifetime, an individual develops an agreement between these basic capacities and secondary capacities, which are internalized with social norms (Secondary Capacities, Table 1). A given unconscious, sustained, intrapsychic response to a prolonged actual conflict can lead to some symptom sequelae: depression, anxiety, psychosomatic disorders, or burnout.

In the present study, we investigated the prevalence of depressive disorders among women during the COVID-19 pandemic and tried to find out possible risk factors responsible for this relationship. We also hypothesized that time and energy distribution in particular areas in women when confronted with the COVID -19 epidemic can be described by a limited number of patterns. Moreover, these patterns may be associated with the risk of depressive symptoms.

## 2. Materials and Methods

### 2.1. Procedure

The presented research results are the part of the results obtained during the implementation of a larger research project about the ways to cope with stress in the context of the COVID-19 epidemic and the effectiveness of psychoeducational strategies (in the field of stress management) based on the principles of transcultural positive psychotherapy. According to its assumptions, a person spends his/her energy in four areas of life. For example, the body dimension includes physical activity, sexuality, nutrition, and relaxation. The dimension of contact comprises interpersonal relations with relatives and the larger social and cultural environment. Productivity or the need to achieve is manifested in taking actions to provide the individuals with a better social status, satisfying work, and development in important areas. The dimension of creativity and fantasy means looking for something beyond the current time. It refers to dreams, future, faith and metaphysics and allows an individual to search for a deeper metaphysical meaning [10]. The part based on the Polish group of respondents will be presented below. Completed surveys were recorded in the survey system and were available only to the respondents. At the end of this step, the data were anonymized by deleting the email addresses. The study lasted from 30 April 2020 to 31 July 2020.

### 2.2. Instruments

The survey was conducted using the questionnaire form on the website. The survey was constructed by 12 experts in the field TPP (“expert judges”), who assigned particular statements to analyzed areas. The concordance of the assignment was 92%. The use of the online survey formula (CAWI) enabled a large number of respondents to be reached during the pandemic. A link to the survey form was sent to the selected persons by e-mail. The survey form included information for the subjects about the purpose and course of the study, a form of voluntary and informed consent of the study participants. The applied exclusion criteria were hospitalization, incapacitation, conscripts, deprivation of liberty, completing the course of psychiatric outpatient treatment and minors. Participants were also informed about ethical aspects such as research confidence and the possibility of withdrawing their answers after they were sent to the survey system via email contact with each of the researchers.

In the part of the research presented in this article, The Beck’s Depression test [12] and the author’s questionnaire were used. It was developed for testing in March 2020 by an international team of trainers (I. Kirillov, P. Meshkova, E. Dobiała). It was created to study the emotional response to the situation associated with COVID-19, as well as the secondary capacities that activate the actual conflict in relation to the four main areas (body, relationships, productivity and creativity). Each of the listed four areas was referred to by 5 questionnaire questions with 5 versions of the answers: “too much”, “more than usual”, “not relevant”, “less than usual” and “not enough”, which were assigned points in the analysis from 5 to 1, respectively. For a particular area, the arithmetic mean was calculated from the scoring of the respective answers. The cut-off point for the occurrence of depressive disorders according to the Beck’s Depression Test was considered to be ≥14 points [13]. The study [14] proved that the Polish version of Beck’s depression scale (BDI-II) is characterized by high reliability; the Cronbach’s alpha for total normalization sample was 0.91, whilst in a group of people with a depression diagnosis, it was 0.93. The authors observed high stability of results: differences between the first and the second round of the test were statistically insignificant, which was demonstrated by a high Pearson’s correlation coefficient (0.86).

### 2.3. Participants

The analysis included 735 women. Demographic data regarding the study population is presented in Table 2.

### 2.4. Data Analysis

**Figure 1:**
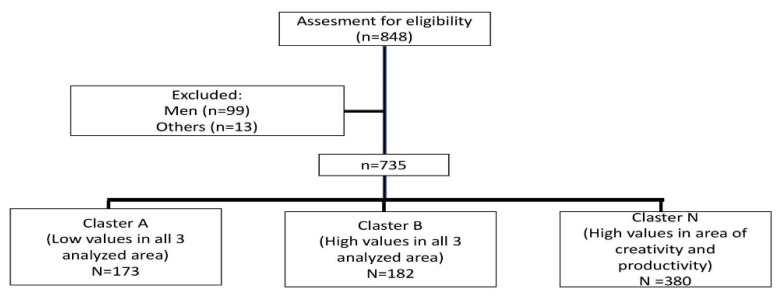
The process of investigation and data analysis.

Due to the skewed distribution of the variables, mainly non-parametric methods were used in the analysis. Data were presented as the median (interquartile range) or number (percent). Differences among groups were tested with the Kruskal–Wallis test (and post hoc two-way test) or Pearson’s chi-square test. The correlation strength of the two variables was expressed with the Spearman’s correlation coefficient. The logistic regression analysis and determination of odds ratio were also performed. A *p*-value < 0.05 was considered statistically significant. The cluster analysis was conducted using standardized values by means of Ward’s method (Euclidean square distance). The cut-off point was chosen based on a tree diagram, presented in Figure S1 in the Supplementary Materials (cut-off point shown with red arrow). Calculations were performed using Statistica version 13, TIBCO Software Inc. Summarizing the process of investigation and data analysis, a flowchart is presented in Figure 1.

## 3. Results

**Figure 2:**
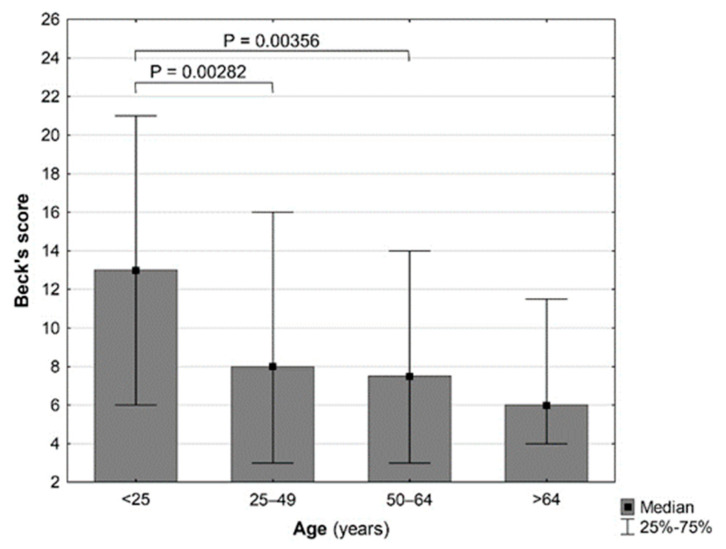
Differences in scoring according to the Beck’s scale in identified age ranges. Statistically significant differences are marked (*P* < 0.05).

The median Beck’s score for the entire study population was 8.00 (3.00–16.00), and in 240 people (32.65%), it suggested the occurrence of depressive disorders (i.e., scores of ≥14). The prevalence of depressive disorders notably depended on the age category of the interviewed people (*P* = 0.00275) and was the highest in the 18–24-year-old age group, in which it reached a frequency of even 47.62% (in comparison with the lowest value of 12.50% in the 50–64-year-old group). Additionally, the presence of a negative correlation between age and score on the Beck’s scale was observed (Spearman R −0.12551, *P* = 0.00065). The median Beck’s scores in different age groups are depicted in Figure 2. Neither the level of education nor the residence influenced the Beck’s score.

There was a correlation between the Beck’s scores and relationship area (Spearman R −0.17445, *P* = 0.00000) and body area (Spearman R 0.31667, *P* = 0.00000) and creativity (Spearman R 0.11148, *P* = 0.002474) but not with productivity area (Spearman R −0.022999, *P* = 0.533581). Correlations were observed between the severities of the different areas (Table 3).

The relationship between body area and Beck’s scores should be interpreted with great caution, as there are many questions (9 out of 21) about the body in the Beck’s questionnaire, and thus, there is a phenomenon of co-linearity. Thus, body area was not included in further analysis.

In order to answer the question about the adaptation strategies acquired by the individuals, a cluster analysis was performed, and three distinct clusters were found (Figure 2):

- Cluster A: low values in all three analyzed areas (relationships, productivity and creativity), including 173 people (23.54% of the entire studied population)
- Cluster B: high values in all three analyzed areas (relationships, productivity and creativity), including 182 people (24.76% of the entire studied population)
- Cluster N: high values in the areas of creativity and productivity but low values in the area of relationships, including 380 people (51.70% of the entire studied population)

No differences regarding age occurred between clusters A, B and N, but significant differences were found in regard to the number of points (*P* = 0.00001) and the prevalence of depression (*P* = 0.00001). These differences are depicted in Table 4.

**Figure 3:**
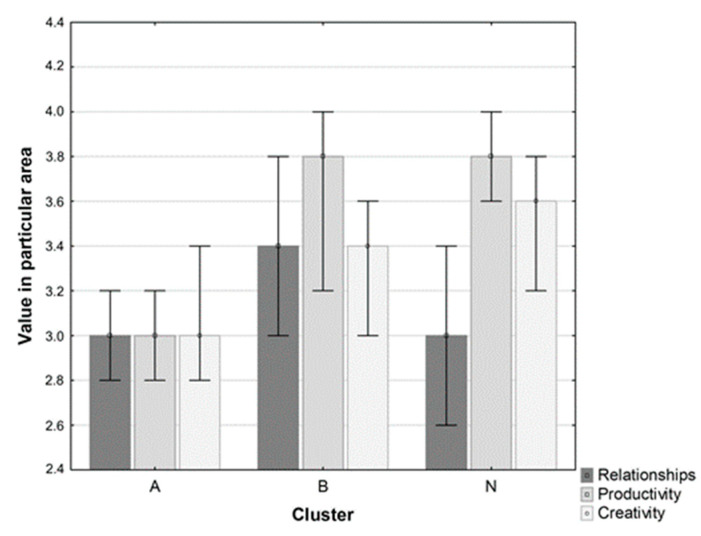
Values (mean) in the analyzed three areas (relationship, productivity, and creativity) in identified clusters. Median (bars) and interquartile range (whiskers) are presented on the graph.

As seen in Figure 3, the relationships variable was responsible for differences between identified clusters. In the multifactor analysis, taking age into consideration, it was established that an increase of the relationships variable by one unit reduced the risk of depressive disorders by half (odds ratio, OR, 0.53; 95% CI: 0.41–0.70; *P* = 0.00001) whereas age < 25 years increased this risk by approximately two-fold (OR 2.22; 95% CI: 1.13–3.46; *P* = 0.00041) (for the entire configuration *P* = 0.00000).

## 4. Discussion

We still do not have conclusive data concerning the impact of the COVID-19 pandemic on mental health, the prevalence of depressive disorders included. The systematic review comprising studies performed until May 2020 found out that during the pandemic, the prevalence of depressive symptoms was 33.7% in the general population [2], which is completely consistent with the results obtained in our survey, where they were found in 32.65% of people. In the recent population-representative, longitudinal study including more than 1100 U.S. adults, the prevalence of depression was 27.8% in 2020 and 32.8% in 2021 [15].

Due to the fact that there are differences between the prevalence of depression among men and women (more common in women) [16], only female individuals were included in this study. The second known, significant variable determining the risk of disorders occurring is the age of the individuals, i.e., the younger the person, the higher the level of depressiveness. In our study, age < 25 years was associated with an over two-fold higher risk of depressive disorders, which was observed in as much as 47.6% of people from that group. Similar findings, indicating that gender and younger age correlate positively with a higher level of depression, were obtained in studies by Fanaj, N. and Mustafa, S. [17], who concluded that the COVID-19 situation had increased the level of depression, especially among women and 18–24-year-olds. Similarly, studies by Lee, J. H., et al. [18] found that depression is higher in women and younger people, aged 20–30-years-old. A meta-analysis including children and adolescents indicated that the global prevalence of depressive and anxiety symptoms is positively correlated with the number of months of the COVID-19 pandemic. Prevalence rates were higher with the increasing age of the child and as a group of women [6]

In contrast, Gao [19] showed that depressive symptoms were more common in the 21–40-year-old group compared to younger people. This study showed that people with higher education were characterized by a lower intensity of depressive symptoms, as well as those from rural areas.

There are many possible explanations for the correlation between age and the risk of depression in the context of the COVID-19 pandemic. The most frequently mentioned in the literature are that they were more likely to use social media, having unlimited access to all kinds of information, including unverified information than older people [11], education level; greater concern for the future; professional stability and greater access to published information in social media [2,19,20,21].

Some studies have shown that the above psychological sequelae may result from social isolation, family financial difficulties, missed development milestones, school disruptions, biological susceptibility, lower baseline self-esteem, a higher likelihood of having experienced interpersonal violence and exposure to stress associated with gender inequity [6,22,23]. Similar conclusions have been drawn by other authors; however, some of them [24,25,26] add that the time of isolation and the pandemic could have been a particularly negatively challenging task for the LGBTQ community because of limiting contact with others from well-known groups.

Besides age, one of the more important factors determining the risk of developing depressive disorders is also the established strategy of coping with a difficult situation. It seems that the model of positive psychotherapy provides an especially valuable tool for explaining the relevant issue. According to the concept of PPT, the distribution of our daily activity in the balance model is formed based on three mechanisms: primary modeling, relational modeling and conscious modelling (at the level of responsibility). In early childhood, in a relationship with a caregiver, basic physiological and emotional needs are met. This provides a basis for the initial modelling of the development of primary capacities (Table 1). The desire to satisfy basic needs and the need for social functioning is at the heart of the emerging personality structure, how intrapsychic conflicts are resolved and the learning of social norms and strategies used in response to stress. When reaching the maturity level that allows for taking responsibility for the conscious development of the ability to independently meet emotional needs and fulfill social norms, the individual is influenced by the awareness of modeling his own model of balance. These dynamics may explain the greater exposure to depressive reactions among younger people who are still developing personality integration [13].

The response to the actual conflict, experienced as stress, can be analyzed from the activation of four dimensions. In the body area, there is an increase in arousal experience as emotions and emotional states. In the area of contact, we pay attention to people to whom we direct our emotions and expectation. In the dynamics of the actual conflict, the emotional stimulation experienced in the body, directed towards others, can lead to the desire to take a certain action and to reveal its results (the area of productivity). As a result, a fantasy area can be triggered: automatic thoughts, fears or images appearing in this area can be experienced.

Depending on the age, the level of integration and developed coping strategies, in situations of stress, the reaction proceeds in a typical way for each individual. There is an escape to a given area of the balance model (a significant part of the response strategy in response to stress comes from a given area) or an escape from a given area (omission of a given area in a stressful situation).

As mentioned above, we hypothesized that time and energy distribution in particular areas in women when confronted with the COVID-19 epidemic could be described by a limited number of patterns and that they may be associated with the risk of depressive symptoms. Indeed, these presumptions were confirmed by results of the present study in the context of the balance model. Results indicated the existence of three types (clusters) of reactions among the interviewed people: (A) avoiding the launch of coping strategies in any area, about ¼ of the population; (B) launching all areas of the balance to adapt to the pandemic situation, about ¼ of the participants; (N) launching strategies from the area of productivity and creativity, bypassing the area of relationships, about ½ of the study group. The effectiveness of the three listed models was varied, and the lowest frequency of depressive disorders was observed in group B.

Besides the effort associated with the coping strategies, the relationships area proved to be a significant variable determining the risk of depressive disorders occurring. In this study we established that making an effort in the relationships area reduces the risk of these disorders by half. It points out that highly developed strategies in the field of productivity (achievements) and creativity (spirituality, fantasy) are not sufficient protective factors in the absence of the possibility of regulation through contact. This is consistent with the conclusions reached by UK researchers [27] that living with others or in the countryside and having closer friends or greater social support were protective factors. Moreover, women experienced it more severely than men, which correlated with higher levels of depression [27]. It is noteworthy that in groups of adolescents declaring healthy relationships at home, the level of depression was lower, which goes along with our research on the preventive role of social contacts in the COVID-19 pandemic [27,28]. It matters that respondents who reported living alone had higher levels of loneliness and depression symptoms than those living with other family members, and this rate was higher in the group of younger people and women [7,28,29].

The results of clinical observations and studies on the mental health of the population conducted in a pandemic situation direct attention to the salutogenetic role of the ability to be in a relationship in a situation of actual conflict. The constraints and pandemic difficulties in this area may become a source of a depression in the female population in the coming years.

These findings are also of practical importance. In order to reduce the risk of depressive disorders developing among women in the COVID-19 pandemic, stimulating development connected to the relationships area should be of fundamental importance. For example, it can be through support or self-development groups.

The Greek studies [21] also examined coping strategies for COVID-19. The researchers measured positive (active) strategies in the study group, which included acceptance, humor, planning, positive reformulation and active coping and supportive (distracting) strategies, which included distraction, ventilation, emotional support and instrumental support. The most common strategies used in both genders were acceptance, humor and planning, so positive strategies. Less often, dysfunctional strategies, such as denial, substance use and withdrawal were used. Supportive and religious coping strategies were more commonly used by women. Higher results in the positive strategy dimension were associated with lower rates of depressive symptoms and anxiety disorders. Similarly, active and passive management in the situation of the pandemic caused by COVID-19 was studied by scientists from China [30]. According to their results, respondents with suspected infection, compared with respondents without suspected infection, used less active coping styles and had lower objective and subjective social support, as well as a lower level of its use. Respondents without suspected infection were more likely to report an active coping style and a higher level of social support, which were protective factors. Thus, the researchers concluded that being single, spending more than 6 h a day searching for COVID-19 information and passive coping styles were risk factors for high levels of mental stress. Non-infected respondents who exhibited high stress levels were more likely to have passive coping styles, such as problem avoidance, fantasy and blaming. Passive coping in these studies has shown a strong association with the worsening of depressive symptoms.

This work has some limitations. First and foremost, it is the lack of a complete psychiatric examination and detection of potential comorbidities, which can affect the intensity of depressive disorders. The second limitation is the uncertainty regarding the true direction of associations between depressive symptoms and the perception of personal relationships, as mood disorders may affect appropriate perception. The third limitation is the methodology, relying on gathering data with the use of an internet questionnaire. The latter was an advantage because it allowed us to analyze the individuals most at risk of this group of disorders occurring, i.e., young people using new technologies.

## 5. Conclusions

This study provides the following, valuable for both psychotherapists and social life moderators, information:

(1) Depressive disorders occur in one-third of women during the COVID-19 pandemic. This risk is even greater at the ages of <25 years old.
(2) Despite the potentially infinite ways of coping with a difficult situation, using the principles of positive transcultural psychotherapy in the analyzed population, three major patterns were successfully isolated, based on the balance model.
(3) Interventions concerning the prevention of mental disorders should primarily target women aged < 25 years old and be directed at strengthening the relationships area, because it is most strongly connected with the risk of depressive disorders occurring.

## Figures and Tables

**Table 1:** Primary and secondary capacities based on [11], modified.

Primary Capacities	Secondary Capacities
Love	Punctuality
Model	Cleanliness
Patience	Orderliness
Time	Obedience
Contact	Politeness
Sexuality	Openness
Trust	Loyalty
Confidence	Justice
Hope	Ambition/Achievement
Faith	Thrift
Doubt	Reliability
Certainty	Exactitude
Unity	Conscientiousness

**Table 2:** Demographic data.

Variable	Mean (±Standard Deviation) or Percentage (Number of Women)
Age	39.61 ± 11.56 years
Education	Higher: 77.82% (*n* = 573) Secondary: 20.28% (*n* = 152) Primary: 1.36% (*n* = 10)
Place of residence	city > 1 mil: 10.75% (*n* = 79) city from 250,000 to 1 mil: 23.54% (*n* = 173) city from 100,000 to 250,000: 13.74% (*n* = 101) city from 50,000 to 100,000: 16.73% (*n* = 123) town < 50,000: 35.24% (*n* = 259)
Current psychotherapy	Yes: 10.20% (*n* = 75) No: 89.80% (*n* = 735)

**Table 3:** Correlations between studied areas. The value of Spearman’s R and the corresponding statistical significance (*P*) are presented.

	Relationships	Body	Productivity
**Body**	−0.06019 NS		
**Productivity**	0.20935 *p* = 0.00000	0.13783 *p* = 0.00018	
**Creativity**	0.15430 *p* = 0.00003	0.30704 *p* = 0.00000	0.53191 *p* = 0.00000

NS—nonsignificant.

**Table 4:** Differences in scoring according to Beck’s scale (median and interquartile range) in identified clusters and differences between them. Additionally, the prevalence of depressive disorders in identified clusters was reported (≥14 points on the Beck’s scale was assumed as the cut-off value).

Cluster	Beck’s Score	Differences between Clusters	Frequency of Depressive Disorders
A	10.00 (3.00–19.00)	A vs. B, *P* = 0.00007 A vs. N, NS B vs. N, *P* = 0.00001	29.17%
B	5.00 (2.00–11.00)		13.75%
N	9.00 (4.50–17.00)		57.08%

NS—nonsignificant.

## Data Availability

The data that support the findings of this study are available on request from the author E.D., Positive Psychotherapy Center in Leszno, pl. Metziga 26, 64-100 Leszno, Poland, edobiala@gmail.com, phone: 48-785877700. The data are not publicly available due to privacy or ethical restrictions.

## Supplementary Materials

The following supporting information can be downloaded at https://www.mdpi.com/article/10.3390/ijerph19127361/s1. Figure S1: cut-off point shown with red arrow.
