# Supplementary material for: Application of the Balance Model in the Analysis of Factors Responsible for Depressive Disorders among Women in the COVID-19 Pandemic
Source: Int J Environ Res Public Health. 2022 Jun 15;19(12):7361. doi: 10.3390/ijerph19127361 (PMC9223622; doi:10.3390/ijerph19127361)
Supplement: Supplementary file 1 [file ijerph-19-07361-s001.zip › ijerph-1699896-supplementary.pdf]

The cut-off point

The cut-off point was chosen based on a tree diagram presented in figure 1 (cut-off point shown with red arrow).

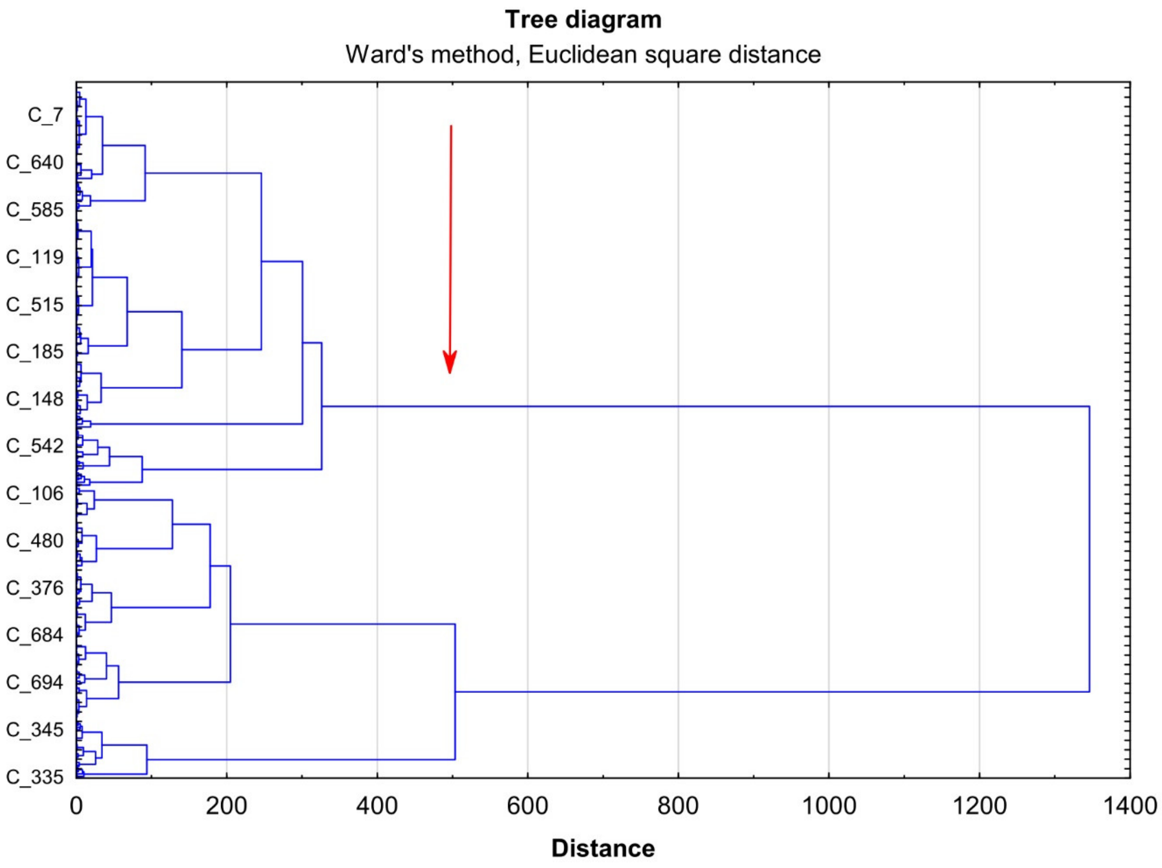

Figure S1: cut-off point shown with red arrow.

The balance model

According to Positive Psychotherapy human life takes places and can be described in four areas: body/senses, achievement, contact, and future/fantasy. In conflicts, every person develops a preference for dealing with the problems in specific area.

Each of the listed four areas was referred to by 5 questionnaire questions with 5 versions of the answers: „too much", „more than usual", „not relevant", „less than usual" and „not enough", which were assigned points in the analysis from 5 to 1, respectively.

Items of the questionnaire:

1. Body, senses:
    - I react emotionally
    - I focus on myself
    - I avoid pain and discomfort
    - I feel paralyzed
    - I watch TV, eat, drink alcohol etc.
2. Achievement, productivity:
    - I check information about the virus
    - I optimize my strategies for actual changes
    - I adjust my expectations to actual changes
    - I actively build my health (exercise, nutrition, discipline, etc.)
    - I optimize my actions to actual changes
3. Contact, relations:
    - I listen to people
    - I cheer everybody up
    - I get support from others
    - I spend time with others
    - I talk to people

4. Creativity, future, fantasy
    - I imagine the worst scenarios
    - I try to understand everything by myself
    - I rely on a Higher Power,
    - I am looking for active, constructive models
    - I optimize my plans according to the actual changes
